# 

**Published:** 1850-02

**Authors:** 


*Buffalo Medical College Commencement.*—The annual commencement in this Institution for the year 1850, will be on Wednesday, the 26th inst. The meeting of Curators for the examination of candidates for graduation, will be on Tuesday, the 25th inst. The valedictory address to graduates will be delivered by Prof. James P. White, and will be given on the afternoon of commencement day.

Medical gentlemen, and others, are cordially invited to be present at the commencement exercises. We hope that the occasion may assemble numerous friends of the College from abroad, as well as at home.

